# A novel allele of the goldfish *chdB* gene: Functional evaluation and evolutionary considerations

**DOI:** 10.1002/jez.b.22831

**Published:** 2018-11-02

**Authors:** Gembu Abe, Ing‐Jia Li, Shu‐Hua Lee, Kinya G. Ota

**Affiliations:** ^1^ Laboratory of Aquatic Zoology, Marine Research Station, Institute of Cellular and Organismic Biology, Academia Sinica Yilan Taiwan; ^2^ Laboratory of Organ Morphogenesis, Department of Ecological Developmental Adaptability Life Sciences Graduate School of Life Sciences, Tohoku University Sendai Japan

**Keywords:** domestication, dorsal–ventral patterning, gene expression patterns, rare allele

## Abstract

The twin tail of ornamental goldfish is known to be caused by a nonsense mutation in one *chordin* paralogue gene. Our previous molecular studies in goldfish revealed that the ancestral *chordin* gene was duplicated, creating the *chdA* and *chdB* genes, and the subsequent introduction of a stop codon allele in the *chdA* gene ( *chdA* ^*E127X*^) caused the twin‐tail morphology. The *chdA* ^*E127X*^ allele was positively selected by breeders, and the allele was genetically fixed in the ornamental twin‐tail goldfish population. However, little is known about the evolutionary history of the *chdB* paralogue, begging the question: are there the functionally distinct alleles at the *chdB* locus, and if so, how did they evolve? To address these questions, we conducted molecular sequencing of the *chdB* gene from five different goldfish strains and discovered two alleles at the *chdB* gene locus; the two alleles are designated *chdB* ^*1*^ and *chdB* ^*2*^. The *chdB* ^*1*^ allele is the major allele and was found in all investigated goldfish strains, whereas the *chdB* ^*2*^ allele is minor, having only been found in one twin‐tail strain. Genetic analyses further suggested that these two alleles are functionally different with regard to survivability ( *chdB* ^*1*^ > *chdB* ^*2*^). These results led us to presume that in contrast to the *chdA* locus, the *chdB* locus has tended to be eliminated from the population. We also discuss how the *chdB* ^*2*^ allele was retained in the goldfish population, despite its disadvantageous function. This study provides empirical evidence of the long‐term retention of a disadvantageous allele under domesticated conditions.

## 1 INTRODUCTION

The *chordin* gene is known to be a major player in dorsal–ventral patterning and axial skeletal formation in vertebrates (De Robertis, 2006, 2009; Inomata et al., 2008; Langdon & Mullins, 2011; Sasai et al., 1994). In fact, it was reported that *chordin* mutant zebrafish (*dino/chordin*) and medaka (*chordin* ^*UT600*^) exhibit ventralized embryonic phenotypes and malformation of the axial skeletal system (Fisher & Halpern, 1999; Schulte‐Merker, Lee, McMahon, & Hammerschmidt, 1997; Takashima et al., 2007). Due to extreme modifications of the developmental program and high lethality in these homozygous *chordin* mutants, genetically stable fixed *chordin* mutant strains have not been found, except for twin‐tail goldfish (*Carassius auratus*; Abe et al., 2014).

The twin‐tail goldfish strain is a well‐established ornamental goldfish strain, which is reared under domesticated conditions (Abe et al., 2014). In our previous molecular developmental study, we found that the twin‐tail goldfish morphology is caused by a stop codon‐containing allele (*chdA* ^*E127X*^) in one of two *chordin* gene paralogues (*chdA* and *chdB*), and the distinct functions of these two paralogues allowed for stable genetic fixation of the phenotype. These paralogues are derived from a lineage‐specific allotetraploidization (the genome duplication of a species hybrid) in the common ancestor of the goldfish and common carp (*Cyprinus caripio*; Ota & Abe, 2016; Luo et al., 2006; Xu et al., 2014), however, the *chdA* ^*E127X*^ allele has only been found in the goldfish lineage. The *chdA* ^*E127X*^ allele lacks three of four functionally significant cysteine‐rich domains, and consequently, its function is highly compromised in comparison with the wild‐type *chdA* allele (*chdA* ^*wt*^). This reduction of function causes a ventralized embryonic phenotype, bilaterally shifted caudal fin primordia, and twin‐tail morphology in the adult (Abe et al., 2014; Abe & Ota 2017). The twin‐tail goldfish was documented in Chinese archives around the 1600s common era (CE), and the origin of goldfish breeding for ornamental purposes dates back to the Song dynasty (around 1000 CE; Chen, 1956; Smartt, 2001); thus, it is expected that the *chdA* ^*E127X*^ allele was selected for and genetically fixed over the course of 600 years, after which it was maintained in the twin‐tail goldfish population. In other words, the *chdA* locus has exhibited a drastic change in the mutated allele frequency under domesticated conditions.

In contrast to the *chdA* locus, the *chdB* locus probably did not experience such a drastic change in allele frequency during the domestication of twin‐tail goldfish, based on its expression patterns and expected function (Abe et al., 2014). The partially overlapping expression patterns of *chdA* and *chdB* genes in embryos suggests that compensation by the *chdB* gene may prevent overreduction of dorsal tissue and increase the survival rate of twin‐tail goldfish without a *chdA* ^*wt*^ allele. Thus, if the loss‐of‐function mutations were to occur at the *chdB* locus in the twin‐tail goldfish, the combined absence of functional *chdA* and *chdB* genes would be expected to lead to high lethality, similar to the reported phenotypes of *dino/chordin* zebrafish and *chordin* ^*UT600*^ medaka (Fisher & Halpern, 1999; Takashima et al., 2007). Consequently, a *chdB*‐mutated twin‐tail goldfish would most likely be eliminated from the population (Fisher & Halpern, 1999; Oelgeschläger, Kuroda, Reversade, & De Robertis, 2003; Schulte‐Merker et al., 1997; Takashima et al., 2007), leading us to presume that the *chdB* locus must be functionally conserved in ornamental twin‐tail goldfish.

However, we still know little about the evolutionary processes acting on the *chdB* gene in goldfish, and we pose several questions, including whether the *chdB* gene has maintained its original function, whether any functionally different alleles exist at the *chdB* locus, and if such alleles exist in the goldfish population, how these alleles have become distributed during the domestication process. To address these questions, we probed for the presence/absence of functionally differentiated *chdB* alleles by examining the *chdB* locus in various goldfish strains and tested how allele variation influences the phenotype of ornamental goldfish. Based on the results of these experiments, we consider how these duplicated paralogues have evolved after the switch from natural to artificial selection.

## 2 METHODS

### 2.1 Goldfish strains

Five different goldfish strains, containing a total of nine subpopulations were used in experiments, including two different groups of the *Butterfly tail* and *Heimutan* strains, *Orandanshishigashira* (*Oranda*), *Ryukin*, and the single‐tail common goldfish strains of Japan, Taiwan, and Mainland China. *Butterfly tail* and *Heimutan* strains were purchased from a local aquarium (Yu‐Dian Corporation) in Yilan, Taiwan. *Oranda* and *Ryukin* strains were purchased from an aquarium fish breeder (SHUEN‐SHIN Breeding Farm) in Toucheng, Taiwan. The single‐tail common goldfish strains from Japan and Mainland China were imported by an aquarium in Taipei, Taiwan (Limpid Aqua/Aqua project Taiwan).

### 2.2 Molecular cloning and phylogenetic analysis

Homologs of *chdB* genes were isolated from complementary DNA (cDNA) derived from embryos of the single‐tail common goldfish and *Oranda* strain using polymerase chain reaction (PCR). Total RNA was extracted from gastrula‐stage *Oranda* goldfish embryos using TRIzol Reagent (Ambion). Specific PCR primers were designed based on the sequence of previously isolated *chdB* genes (accession number: BAO51897). Amplified PCR fragments were isolated and purified and then ligated into a vector using the TOPO TA Cloning Kit, Dual Promoter (Invitrogen). The resulting vector was used to transform DH5α *Escherichia coli*. More than 10 clones were picked for sequencing. The sequences of the cDNA fragments were then used as backbones to obtain nearly complete sequences of the alleles by PCR with specific primers. The isolated gene was identified by generating multiple amino acid alignments with known goldfish, orthologous and paralogous genes using CLUSTALW. The phylogenetic relationship of *chdB* ^2^ alleles and closely related *chordin* genes was reconstructed in a maximum likelihood tree using MEGA5. Branch lengths were estimated based on the nonsynonymous and synonymous substitutions using PAMLX (Xu & Yang, 2013).

### 2.3 Goldfish breeding for segregant analysis

Total 21 individuals from the *Oranda* strain were genotyped by PCR amplification and restriction digestion. Of those individuals, four individuals having a heterozygous locus of *chdB* (designated as OR7M, OR9F, OR18M, OR24F) were used for breeding. In 2012, OR7M was crossed with OR24F, and OR18M was crossed with OR9F to obtain the F1 generation. F1 goldfish were tracked by a microchip tagging system (MUSICC Identification System and MiniTracker I, Avid). From the F1 population, six individuals were used to produce the F2 generation. In 2015 and 2016, F2 segregants were obtained from the F1 population. Artificial fertilization was performed according to methods detailed in our previous report (Tsai, Chang, Liu, Abe, & Ota, 2013).

### 2.4 Genotyping

PCR primers were designed to amplify a region containing four SNP sites and a *Cla* I restriction enzyme site. PCR fragments amplified by these specific primers were digested by *Cla* I, and separated on 2% agarose gels. Genotypes were determined on the basis of the resulting band patterns. The band patterns were confirmed by at least two independent trials of PCR and restriction enzyme digestion.

### 2.5 Injection of messenger RNA

To generate constructs for transcription, the coding regions of *chdB* ^*1*^ or *chdB* ^*2*^ were amplified by PCR and cloned into the pCS2+vector (Rupp, Snider, & Weintraub, 1994). These constructs were first digested with *NotI* and then used as templates to synthesize capped messenger RNA (mRNA) with the mMESSAGE mMACHINE SP6 Kit, according to the manufacturer’s instructions (Ambion). The synthesized mRNA transcripts were purified with Quick Spin Columns and resuspended in nuclease‐free water. A microinjector (Eppendorf Femtojet; Eppendorf, Hamburg, Germany) was used to inject mRNA into the yolk of one to two cell‐stage fertilized eggs in 4 nl of 0.2 M KCl. Phenol red (Sigma) was added as an indicator at a final concentration of 0.05%. In total, 100 pg of mRNA was injected into *chdB* ^*1/1*^ twin‐tail goldfish embryos. The injected embryos were incubated at 24°C. Four independent rescue experiments were performed by injecting *chdB* ^*1*^ or *chdB* ^*2*^ mRNA into twin‐tail goldfish embryos. Control embryo phenotypes were examined in all four experiments. All control embryos exhibited mutant phenotypes. Two days after injection, embryos were classified by morphological inspection into the following four categories: dorsalized, weakly ventralized, bifurcated fin fold, and severely ventralized. The categorization was based on our previous report (Abe et al., 2014). To investigate the larval morphology of injected individuals, larvae were anesthetized with MS222 two days after injection and photographed live and/or after fixation with 4% paraformaldehyde (PFA). The fixed larvae were stained by alizarin red solution (0.02% alizarin red in 70% ethanol) and examined under a stereomicroscope (SZX16; Olympus, Tokyo, Japan).

### 2.6 In situ hybridization

Digoxigenin‐labeled antisense RNA probes were produced using PCR product templates and the T7 RNA polymerase Riboprobe Combination System (Promega), according to the manufacturer's instructions. The probes were purified using mini Quick Spin RNA Columns (Roche, Germany). Primer sets for the PCR amplification of *szlA* and *foxb1a* fragments were from a previous report (Abe et al., 2014). PCR products corresponding to relevant portions of 5′‐ or 3′‐untranslated regions were used to generate probes.

Whole‐mount in situ hybridization was performed as previously described (Schulte‐Merker, Ho, Herrmann, & Nusslein‐Volhard, 1992) with minor modifications. Fish embryos were fixed with 4% paraformaldehyde in phosphate‐buffered saline (PBS) overnight. Embryos were fixed and then dechorionated using fine forceps. After fixation and dechorionation, embryos were dehydrated with methanol. Dehydrated embryos were then rehydrated with phosphate‐buffered saline, 0.1% Tween‐20 (PBT) and fixed with 4% paraformaldehyde in PBS. Embryos were subsequently treated with proteinase K for 20 min, after which the samples were fixed again. Prehybridization and hybridization were performed at 65°C for a period that ranged between 1 hr and overnight. The samples were washed sequentially two times with 50% formamide/2 × SSCT at 65°C for 30 min, 2 × SSCT at 65°C for 15 min, and two final washes with 0.2 × SSCT at 65°C for 30 min. The samples were then incubated in blocking solution, consisting of 10% heat‐inactivated goat serum (Roche, Germany) and 0.1% Tween‐20 in PBS, for 1 hr, before being incubated with a 1:4,000–8,000 dilution of anti‐digoxigenin‐AP Fab fragments (Roche, Germany) at room temperature for 4 hr, or at 4°C overnight. Samples were washed four times with blocking solution at room temperature for 25 min each. Signals were detected using BCIP/NBT Color Development Substrate (Promega). The reaction was stopped by washing samples with 20% MeOH in PBS. To ensure an accurate comparison of gene expression levels, the embryos in a single experiment were treated at the same time under identical conditions.

### 2.7 Measurement of the *szlA*‐expressing area

Embryos that were labeled for *szlA* mRNA expression were placed on a 0.5% agarose plate and photographed from the left lateral view. To minimize bias from lighting, the position of the light source was adjusted while monitoring the light intensity in the active live image with the *line profile function* in the cellSens software (Olympus). The maximal length from the most ventral to the most dorsal boundary of the *szlA* mRNA positive area (szl.DV), the maximal diameter of the embryo along the dorsal–ventral axis (emb.DV), and the maximal diameter of the blastopore along the dorsal–ventral axis (bp.DV) from the lateral view were measured with Image J software. The proportions of *szlA*‐positive area and blastopore closure were calculated as szl.DV/emb.DV × 100 and (emb.DV − bp.DV)/bp.DV × 100, respectively.

### 2.8 Morphological analyses of bifurcated anal and caudal fins

Goldfish larvae from Asb to Pr stage were anesthetized with MS222 (Sigma), and then fixed with 4% paraformaldehyde in PBS. After fixation, samples were washed in 70% ethanol, stained with alizarin red solution (0.02% alizarin red in 95% ethanol), and washed again in 70% ethanol to reduce background (Li, Chang, Liu, Abe, & Ota, 2015). The number of the caudal fin rays were counted under a stereomicroscope (SZX16; Olympus). All goldfish specimens that were used for morphological analysis were genotyped at the *chdB* locus and categorized into three groups according to their allelic combination: *chdB* ^*1/1*^, *chdB* ^*1/2*^, and *chdB* ^*2/2*^.

### 2.9 Statistical analyses

All plotting and statistical tests in the morphological and genetic analyses were performed with the R statistical computing package of RStudio v0.98.1049.

## 3 RESULTS

### 3.1 Molecular background of two *chdB* alleles in goldfish

**Figure 1:**
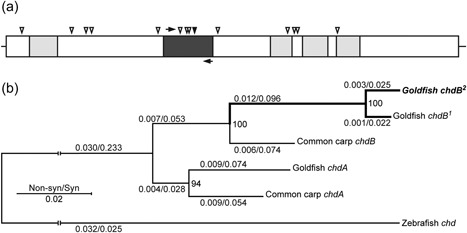
Molecular characteristics of the *chdB* gene in goldfish. (a) Schematic view of the cDNA sequence of the *chdB* gene. Light gray boxes indicate cysteine‐rich (CR) domains. Dark gray box indicates exon 11. Black arrows indicate binding sites for PCR primers to amplify the 334 bp of DNA fragment used for genotyping. The forward and reverse primers are chdb‐gf1 (5′‐AGTGGGGTCGGCTGTCTTCACT‐3′) and chdb‐gr1 (5′‐ATCCATTCCGTTGTACGGCAGCATTT‐3′). Arrowheads indicate nonsynonymous SNP sites. The nonsynonymous SNP indicated by the black arrowhead is recognized by *ClaI* restriction enzyme. (b) Phylogenetic tree of chordin gene sequences from three Cypriniformes species, comprising the *chdA* and *chdB* genes of goldfish (*Carassius auratus*) and common carp (*Cyprinus carpio*), with the zebrafish chordin gene (*Danio rerio*) as an outgroup. The topology of the phylogenetic relationships was inferred by 934 amino acid residues. Branch lengths based on substitutions are indicated on each branch (nonsynonymous/synonymous). The bootstrap value of maximum likelihood analysis are shown in the nodes. cDNA: complementary DNA; PCR: polymerase chain reaction

Our molecular cloning of *chdB* genes revealed that two different alleles exist in the *Oranda* population. One allele was first found in our previous report (accession number: BAO51897), and the other is a newly discovered allele (LC382263); the two alleles were named *chdB* ^*1*^ and *chdB* ^*2*^, respectively (Figure 1a). A phylogenetic tree of *chdB*‐related sequences, which was created based on maximum likelihood analysis, suggested that these two allele sequences diverged from each other (Figure 1b). The estimated ratios for the numbers of nonsynonymous/synonymous substitutions per site are 0.045 and 0.120 for the *chdB* ^*1*^ and *chdB* ^*2*^ branches, respectively. From pairwise comparisons of 2,787 nucleotide sites in the coding regions of these *chdB* alleles, 42 single‐nucleotide polymorphisms (SNPs) were found, 14 of which are nonsynonymous (Figure 1a). Two nonsynonymous SNPs were located on the cysteine‐rich domain, which is highly conserved among all vertebrate species (Garcia Abreu, Coffinier, Larraín, Oelgeschläger, & De Robertis, 2002), implying that these two SNPs might influence to the function of *chdB* gene. In addition, one of four nonsynonymous sites in exon 11 is recognized by the *ClaI* restriction enzyme, allowing us to develop a *ClaI*‐based genotyping method.

To investigate whether the *chdB* ^*1*^ allele is a major or minor allele in the ornamental goldfish population, we used PCR amplification and *ClaI* restriction enzyme digestion to genotype 102 goldfish, comprising five different strains and nine subpopulations (Figure 1a and Table 1). Among the genotyped fish, only those from the *Oranda* strain carried the *chdB* ^*2*^ allele (Table 1). The allele frequency for *chdB* ^*2*^ was 0.214 in the *Oranda* strain and 0.044 across all investigated strains, suggesting that the *chdB* ^*2*^ allele is minor allele in the overall ornamental goldfish population.

**Table 1:** Genotypes of ornamental goldfish strains

Strain (subpopulation ID)	*chdB* ^*1/1*^	*chdB* ^*1/2*^	*chdB* ^*2/2*^	Total
Butterfly tail (20151124)	10	0	0	10
Butterfly tail (20150707)	14	0	0	14
Heimutan (20151124)	16	0	0	16
Heimutan (20150707)	12	0	0	12
Oranda (YiMS‐Taiwan)	12	9	0	21
Ryukin (YiMS‐Taiwan)	4	0	0	4
Single fin^*^ (YiMS‐Japan)	11	0	0	11
Single fin^*^ (YiMS‐China)	5	0	0	5
Single fin^*^ (YiMS‐Taiwan)	9	0	0	9
Total	93	9	0	102

^*^ Single fin common goldfish strain.

### 3.2 Functional analyses of allelic differences of *chdB* gene

**Figure 2:**
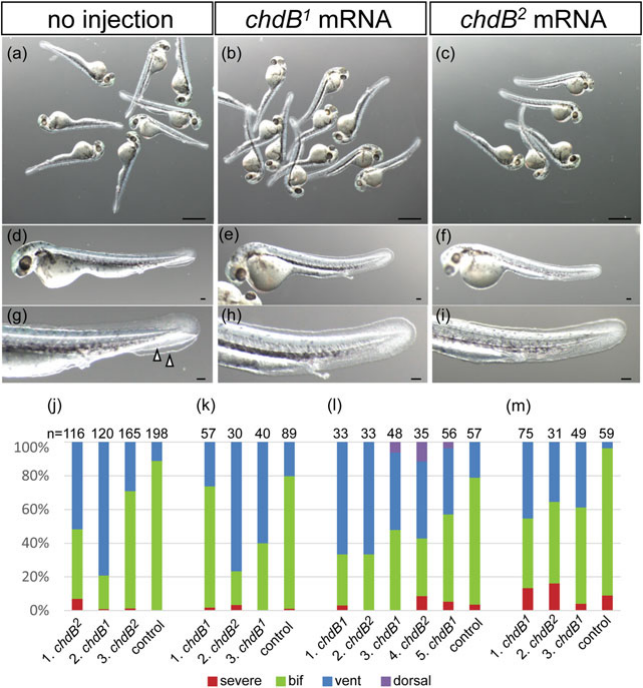
Phenotype rescued individuals of goldfish and zebrafish chordin gene mutants. Larval phenotypes of twin‐tail goldfish. Noninjected controls (a), embryos injected with *chdB* ^*1*^ (b), or *chdB* ^*2*^ mRNA (c). Representative individuals of noninjected controls (d) and embryos injected with *chdB* ^*1*^ (e) or *chdB* ^*2*^ mRNA (f). (g–i) Magnified views of the caudal regions in d–f. White arrowheads indicate bifurcated caudal fin. (j–m) Proportion of specimens with different phenotypes, after injection of embryos with mRNA in four independent experiments. The number of larvae analyzed is indicated above each bar. The order of the injection is indicated by labels on the horizontal axis. Scale bars = 1 mm (a–c), 0.1 mm (d–i). mRNA: messenger RNA [Color figure can be viewed at wileyonlinelibrary.com]

**Figure 3:**
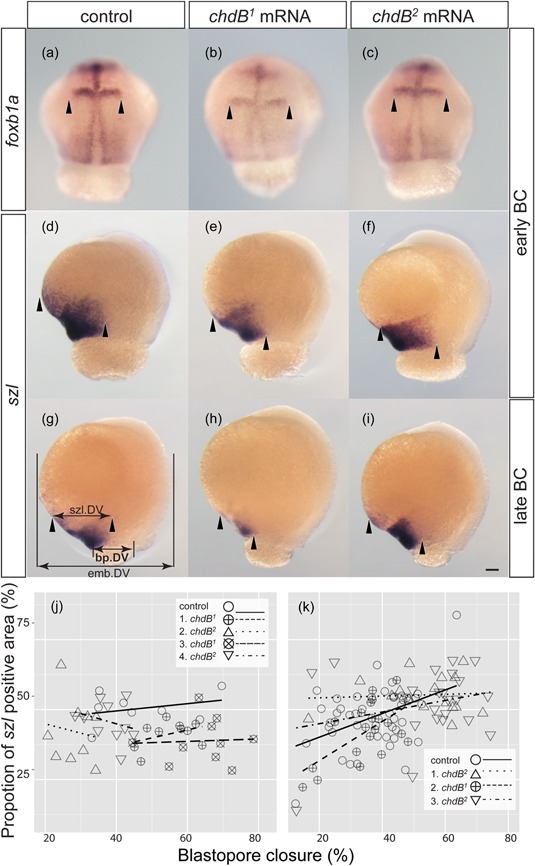
Gene expression patterns of *foxb1a* and *szl*. Expression patterns of *foxb1a* in control (a), and *chdB* ^*1*^‐ (b), or *chdB* ^*2‐*^mRNA‐injected twin‐tail goldfish embryos (c). Dorsal views of gastrula‐stage embryos (50% blastopore closure) are shown. (d–i) Expression patterns of *szlA* in early blastopore closure stages (d–f) and late blastopore closure stages (g–i). Embryos are shown from lateral view. The left (d,g), middle (e,h), and right (f,i) panels represent control, *chdB* ^*1*^‐ and *chdB* ^*2*^‐mRNA‐injected embryos, respectively. (j,k) Plots of the relationship between developmental stage and *szl* gene expression patterns in the first (j) and second (k) experiments. Injected mRNA groups are represented by circles (control), crossed circles (*chdB* ^*1*^), circles with X (*chdB* ^*1*^), triangles (*chdB* ^*2*^), and inverted triangles (*chdB* ^*2*^). The order of the mRNA injection is represented by prefix numbers in the key displayed on each graph (j,k). Solid, dashed, long‐dashed, dotted, and dot‐dashed lines represent fitted trends. The fitted lines were estimated by a generalized linear model with quasi‐likelihood estimation. szl.DV, emb.DV, and bp.DV indicate the maximum distance of the *szl* gene‐expressing region along the dorsal–ventral axis, diameter of the embryo along the dorsal–ventral axis, and diameter of blastopore closure along the dorsal–ventral axis, respectively. Panels (a–i) are of the same magnification. Scale bar = 0.1 mm (i). bp.DV: blastopore along the dorsal–ventral axis; emb.DV: embryo along the dorsal–ventral axis; mRNA: messenger RNA; szl.DV: *szlA* mRNA positive area [Color figure can be viewed at wileyonlinelibrary.com]

Next, we conducted mRNA microinjection rescue experiments to examine whether there are functional differences between the *chdB* ^*1*^ and the newly discovered *chdB* ^*2*^ alleles at different developmental stages (Figures 2 and 3). We injected the same amount (100 pg/embryo) of *chdB* ^*1*^ or *chdB* ^*2*^ mRNA into *chdB* ^*1/1*^ twin‐tail goldfish embryos (Figure 2). The embryonic phenotypes were then examined at the prehatching stage when the primordia of the caudal fin (caudal fin fold) can be observed (Figure 2a–i). Proportions of weakly ventralized or dorsalized embryos in both *chdB* ^*1*^‐ and *chdB* ^*2*^‐mRNA‐injected goldfish were higher than those in uninjected embryos (Figure 2j–m), suggesting that both alleles function as dorsal organizers, similar to other vertebrate *chordin* gene orthologues (Figure 2; Abe et al., 2014; Inomata, Haraguchi, & Sasai, 2008; Takashima et al., 2007; De Robertis, 2006, 2009; Langdon & Mullins, 2011). However, we could not detect any differences between *chdB* ^*1*^ and *chdB* ^*2*^ in the phenotypes of mRNA‐injected embryos at this stage, presumably due to subtle or a lack of functional differences at the stages we examined.

Because we expected that differences between *chdB* ^*1*^‐ and *chdB* ^*2*^‐mRNA‐injected embryos may be most clearly detected in early‐stage embryos, we probed the gene expression patterns of *foxb1a* (an embryonic neural marker expressed in dorsal cells) and *szlA* (an embryonic ventral marker) at two different gastrula stages (Figure 3). For *foxb1a*, differences in gene expression patterns between controls, *chdB* ^*1*^‐, and *chdB* ^*2*^‐mRNA‐injected embryos were subtle (Figure 3a–c). On the other hand, the *szlA* gene showed markedly reduced expression in early‐to‐late gastrula‐stage *chdB* ^*1*^‐ and *chdB* ^*2*^‐mRNA‐injected embryos compared with controls (Figure 3d–i). Importantly, the reduction in *szlA* gene expression was more severe in *chdB* ^*1*^‐mRNA‐injected embryos than *chdB* ^*2*^‐mRNA‐injected embryos at both early and late gastrula stages (Figure 3e,f,h,i).

In addition to visual inspection (Figure 3a–i), quantification of the *szlA* gene expression region was performed (Figure 3j,k). We measured the *szlA* gene expression area in *chdB* ^*1*^‐ or *chdB* ^*2*^‐mRNA‐injected goldfish embryos that were derived from two different clutches across multiple developmental stages, ranging from more than 20% blastopore closure to less than 80% blastopore closure (Figure 3g). All of the *chdB* ^*1*^‐mRNA‐injected embryos showed narrower *szlA* gene expression areas in comparison with controls (Figure 3j,k), consistent with our previous report (Abe et al., 2014). On the other hand, the *chdB* ^*2*^‐mRNA‐injected embryos tended to exhibit expanded *szlA* gene expression areas in comparison with *chdB* ^*1*^‐mRNA‐injected embryos, even though we injected the same amount of mRNA, and the twin‐tail goldfish embryos for each experiment were derived from the same clutch (Figure 3j,k). Because the same trend was observed in multiple experiments after different orders of injection, the differences might reflect actual differences in the function of the two alleles (Figure 3j,k). These results suggested that *chdB* ^*1*^ and *chdB* ^*2*^ both function as dorsal organizers, however coding‐region‐localized SNPs may affect *szlA* gene expression patterns in early embryonic stages (Figures 1 and 3).

### 3.3 Genotyping and morphological analyses in F2 segregants from *chdB* ^*1/2*^ parents

**Figure 4:**
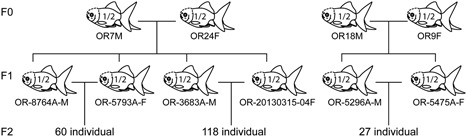
Pedigree of goldfish. In total, 205 F2 segregants derived from 4 parents were analyzed. The genotype of each F0 and F1 individual is indicated in goldfish illustration; “1/2” indicates *chdB* ^*1/2*^. Six F1 individuals were designated as OR‐8764A‐M, OR‐5793A‐F, OR‐3683A‐M, OR‐20130315‐04F, OR‐5296A‐M, and OR‐5475A‐F (M and F indicate male and female, respectively)

**Figure 5:**
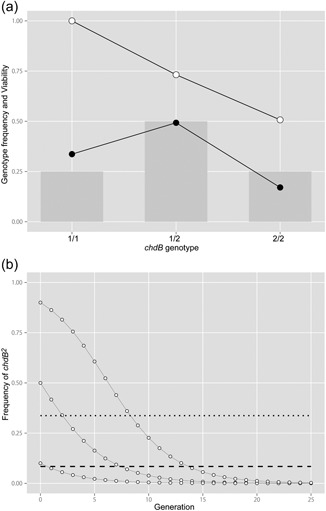
Viability and theoretical *chdB* ^*2*^ allele frequency in ornamental goldfish. (a) Genotype frequency and viability of the segregants derived from *chdB* ^*1/2*^ *Oranda* goldfish parents. Light gray bars indicate the theoretical allele frequency based on Mendelian predictions (*chdB* ^*1/1*^:*chdB* ^*1/2*^:*chdB* ^*2/2*^ = 1:2:1). Solid circles indicate the observed genotype frequency in the segregant population. Open circles show the viability calculated from the observed genotype frequency. (b) The theoretical expectation of allele frequency across generations is based on the relative fitness of each genotype using the generation‐by‐generation algorithm (open circles with fine solid lines; Hartl & Clark, 1997). The initial allele frequencies are 0.9, 0.5, and 0.1. The observed allele frequency in the *Oranda* goldfish strain population is indicated by a dotted line, and the average allele frequency amongst all investigated goldfish populations is indicated by a dashed line

To further clarify whether *chdB* ^*1*^ and *chdB* ^*2*^ differentially influence the phenotypes of twin‐tail goldfish, we crossed *chdB* ^*1/2*^ parents of the *Oranda* strain, and subsequently examined the genotypes of the progeny at the juvenile stage (Ar stage). At this stage, almost all of caudal fin rays are countable (Li et al., 2015). In total, 205 individuals were observed from three different crosses (Figure 4 and Table 2). The numbers of progeny with *chdB* ^*1/1*^, *chdB* ^*1/2*^, and *chdB* ^*2/2*^ genotypes were 69, 101, and 35, respectively. Therefore, the ratio of genotypes (0.337:0.493:0.170) deviated from the expected 0.25:0.50:0.25 Mendelian ratio (*p* < 0.01, the chi‐square test; Table 2 and Figure 5a). Since all of the fish were kept under identical conditions, it is expected that the minor deviation from Mendelian ratios reflects viability differences between the genotypes. Thus, the allelic variants in *chdB* may influence phenotypic features related to lethality (e.g., early embryogenesis and/or physiological features). From the genotype frequency of *Oranda* F2 segregants, we also estimated the fitness of *chdB* genotypes relative to *chdB* ^*1/1*^ (Figure 5a). The relative fitness estimates for *chdB* ^*1/1*^, *chdB* ^*1/2*^, and *chdB* ^*2/2*^ were 1.000, 0.732, and 0.507, respectively, suggesting additive gene action (solid circles, Figure 5a).

**Table 2:** Genotypes of F2 progeny

Strains	Male	Female	*chdB* ^*1/1*^	*chdB* ^*1/2*^	*chdB* ^*2/2*^	Total
1	OR‐8764A‐M	OR‐5793A‐F	21	29	10	60
2	OR‐3683A‐M	OR‐20150315‐04F	39	60	19	118
3	OR‐5296A‐M	OR‐5475A‐F	9	12	6	27
Total	3 individual	3 individual	69	101	35	205

On the basis of these relative fitness estimates, we calculated the theoretically expected change in the frequency of the *chdB* ^*2*^ (Figure 5b). According to this analysis, the frequency of the *chdB* ^*2*^ allele is expected to be less than 0.001 at the 21st generation, even when the allele frequency of the initial population is 0.9 in a random mating model (Hartl & Clark, 1997; Hedrick, 2005). The domestication history of twin‐tail goldfish spans more than 400 years, and two‐ to four‐year‐old goldfish are optimal for obtaining the next generation of offspring (Matsui, 1972). Thus, ornamental goldfish populations have probably been maintained for at least 100–200 generations. In light of the expected quick decline in *chdB* ^*2*^ frequency under a random mating model, the *chdB* ^*2*^ allele should have already been completely eliminated from the ornamental goldfish population (Figure 5b). Persistent retention of *chdB* ^*2*^ allele in the *Oranda* population prompted us to ask whether the *chdB* ^*2*^ allele contributes to the attractiveness of caudal fin morphology, as has been observed for the *chdA* locus.

**Figure 6:**
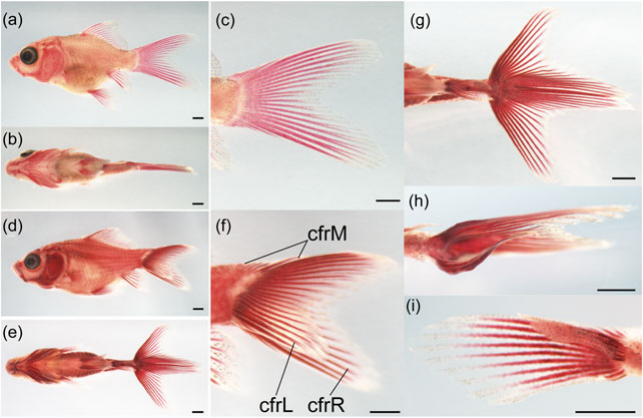
Representative caudal skeletal morphology of F2 segregants. (a–c) Goldfish with a single caudal fin. (d–i) Bifurcated caudal fin individuals. (a,d) Lateral view of the whole body. (b,e) Ventral view of the whole body. (c,f,g) Magnified view of caudal regions of panels a,d, and e. (h,i) Two representative examples of the asymmetrically bifurcated caudal fin. The caudal fin ray number in nonbifurcated caudal fin rays (cfrM) is 7. The left‐side bifurcated caudal fin ray number (cfrL) is 23, and the right‐side bifurcated caudal fin ray number (cfrR) is 29 in the individual shown in panel h. The cfrM, cfrL, and cfrR are 4, 11, and 7, respectively, for the individual shown in panel i. Scale bars = 1 mm. Panels a–d are of the same magnification [Color figure can be viewed at wileyonlinelibrary.com]

To more closely investigate the caudal fin morphology, we first categorized all of the F2 segregants as either single caudal fin or bifurcated caudal fin types (Figure 6a–f; Supporting Information Table S1); more than 90% of the segregants (192 individuals) had bifurcated caudal fins, which varied in their symmetricity (Figure 6g,h,i). Most of the segregants had a highly symmetrical bifurcated caudal fin (Figure 6g), but a few of the fish exhibited an asymmetrically bifurcated caudal fin, with one lobe being larger than the other (Figure 6h,i).

To distinguish whether these morphological variations were correlated with the *chdB* allele, we counted the number of caudal fin rays in the 205 individual segregants. Caudal fin rays were counted and categorized as “left‐side bifurcated caudal fin rays” (cfrL), “right‐side bifurcated caudal fin rays” (cfrR), and the “midline located nonbifurcated caudal fin rays” (cfrM; Figure 6f). The summations of cfrM and cfrL as well as cfrM and cfrR were calculated, and the larger sum was used as an index of the maximal number of caudal fin rays along the dorsal–ventral axis; this index is designated as the “maximum caudal fin number.”

**Figure 7:**
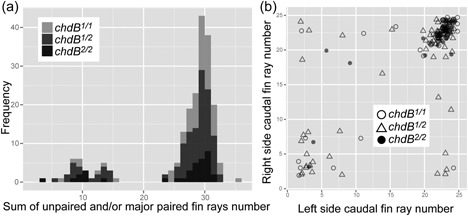
The relationship between genotype and caudal fin morphology. The histogram shows the distribution of the sum of the caudal fin rays in the medial portion and left (or right) side (cfrM+max(cfrL, cfrR)). The distributions of caudal fin ray numbers are highly deviated from normal distributions (Shapiro–Wilk test, *p* < 0.001). (b) The relationship between left and right fin ray numbers. The open circles, triangles, and closed circles represent *chdB* ^*1/1*^, *chdB* ^*1/2*^, and *chdB* ^*2/2*^, respectively. Spearman's correlation coefficients of *chdB* ^*1/1*^, *chdB* ^*1/2*^, and *chdB* ^*2/2*^ are 0.631, 0.580, and 0.678 (*p* < 0.01), respectively. The sample number and ratio of the genotypes are described in Supporting Information Table S1. cfrL: left‐side bifurcated caudal fin rays; cfrM: midline located nonbifurcated caudal fin rays; cfrR: right‐side bifurcated caudal fin rays

The distribution of the maximum caudal fin number indices was divided into two phenotypic groups (Figure 7a). The F2 progenies of 33 individuals had an index less than 20, whereas 172 individuals had an index of more than 20 (Figure 7a); we categorized those less than 20 as the “less caudal fin ray number” group and those more than 20 as the “normal caudal fin ray number” group (Figure 7a). The total numbers of fin rays in the normal caudal fin number group was almost equivalent with single‐fin common goldfish at the equivalent stage (Li et al., 2015); single‐fin common goldfish exhibit 30 caudal fin rays at the juvenile stage, whereas the F2 progeny in the normal caudal fin ray number group have an average of 29 fin rays (Figure 7a). After distinguishing the fish phenotypes according to this scheme, we could not detect any significant relationship between allelic combinations and the maximum caudal fin numbers (Supporting Information Table S2).

We also plotted left and right fin ray number of the 192 individuals with bifurcated caudal fins, as shown in Figure 7b. Although there are variations, our analyses suggested that the number of left and right fin rays in the caudal fin is correlated to each other in all of the genotypes (Figure 7b). Moreover, the *chdB* ^*2/2*^ genotype progenies exhibit only a faintly higher correlation in left‐right caudal fin ray numbers than the progenies with the other two genotypes (Figure 7b). These results led us to conclude that the *chdB* ^*2*^ allele does not significantly influence visually recognizable caudal fin morphology, which may be favorably selected by conventional breeders. However, our result does imply that the *chdB* ^*2/2*^ genotype slightly reduces the number of individuals with extremely asymmetric caudal fin morphology (Figure 7b).

## 4 DISCUSSION

Our molecular sequencing and developmental analyses of the *chdB* locus revealed the presence of two functionally differentiated alleles, *chdB* ^*1*^ and *chdB* ^*2*^ (Figure 1). Moreover, the genotyping of various goldfish populations suggested that the *chdB* ^*2*^ allele is a minor allele across goldfish populations. This low allele frequency of *chdB* ^*2*^ in the investigated goldfish populations is consistent with the additive reductions in viability for *chdB* ^*2*^ allele copies in *Oranda* lab strain progeny (Table 2 and Figures 4 and 5). On the basis of minor differences between *chdB* ^*1*^ and *chdB* ^*2*^ mRNA‐microinjected *chdA* ^*E127X/E127X*^ embryos (Figures 2 and 3), it is reasonable to conclude that the low survival rates of *chdB* ^*1/2*^ and *chdB* ^*2/2*^ genotypes might be caused by deleterious events at later developmental stages. In addition, our analyses do not show an obvious relationship between the genotype and caudal fin morphology (Figures 6 and 7; Supporting Information Tables S1 and S2). These results illustrate a major difference between selective pressures on the *chdA* and *chdB* loci. The *chdA* ^*E127X*^ allele, which almost completely lacks function, was positively selected during the domestication process, based on its twin‐tail morphology phenotype. On the other hand, the *chdB* locus might have been eliminated from the population, because it does not seem to contribute to any preferred morphology. Thus, it is reasonable to presume that the *chdB* ^*2*^ allele has been eliminated from most goldfish populations during the domestication process, as would be predicted by the random mating and selection model (Hartl & Clark, 1997; Hedrick, 2005; Figure 5b).

However, the random mating and selection model does not explain our finding that the *chdB* ^*2*^ allele is still present in goldfish populations at least 100 generations after its fixation approximately 400 years ago. Under the random mating model, the *chdB* ^*2*^ allele will be almost eliminated from the population by the 25th generation due to its disadvantageous nature, even if the allele were initially a major allele (Figure 5B). These results lead us to pose the question of how the *chdB* ^*2*^ allele is still retained in the modern ornamental goldfish population.

First, stochastic factors should be examined to test whether these mechanisms explain the retention of the *chdB* ^*2*^ allele in the *Oranda* goldfish population. For example, there is a possibility that some ornamental goldfish subpopulations carry the *chdB* ^*2*^ allele at a very high frequency, but this subpopulation was absent from our investigated fish populations. In fact, the ornamental goldfish population tends to be divided into small segregated subpopulations due to convenient maintenance in small ponds and aquarium tanks (Matsui, 1972). Thus, genetic drift may have occurred to cause the fixation of the *chdB* ^*2*^ allele in a goldfish subpopulation (Hartl & Clark, 1997; Hedrick, 2005). The existence of such a subpopulation might explain the retention of the *chdB* ^*2*^ allele in the overall goldfish population. To examine this assumption, extensive genotyping of the *chdB* locus should be performed in a wide variety of *C. auratus* populations, including ornamental goldfish and wild‐type crucian carp, which represent an outgroup of the ornamental goldfish population (Komiyama et al., 2009; Takada et al., 2010).

In addition, it should be considered that the *chdB* ^*2*^ allele could be advantageous on a certain genetic background and/or in certain environments. For example, subtle differences in the numbers of the left and right caudal fin rays between the three *chdB* genotypes may suggest that these differences may be less subtle under other circumstances (Figure 7b). The twin‐tail goldfish with the *chdB* ^*2/2*^ genotype showed a slightly lower proportion of individuals with extreme asymmetry in the twin‐tail morphology (Figure 7b). Therefore, we may speculate a possibility that this enforcement of symmetry could be enhanced under the different genetic backgrounds or environmental conditions. In such a case, the retention of the *chdB* ^*2*^ allele would be explainable by positive selection of symmetric twin‐tail morphology for the ornamental purposes, as discussed by Darwin (1868). Because it is known that genetic background and/or environments influence phenotypes in several animals (Gilbert & Epel, 2009; Waddington, 1957), it would be worthwhile to examine whether the expressivity of the *chdB* ^*2*^ allele could be altered by applying different genetic backgrounds and/or environments. Such an experiment would likely require the creation of hybrids between various different types of ornamental goldfish strains with different morphologies, and raising the hybrids under various environmental conditions (Smartt, 2001).

We also discuss several remaining problems to be addressed in future studies. Our current study suggests that the divergence and contrasting evolutionary processes observed for goldfish *chdA* and *chdB* paralogues are due to the relative contributions of these genes to the twin‐tail morphology under domesticated conditions. On the other hand, our previous study reported a conservative evolutionary process for the *chdA* and *chdB* genes in common carp, based on their gene expression patterns. Moreover, an equivalent stop codon allele for *chdA* has not been found in our previous studies or publicly available genomic data for the common carp (Abe et al., 2014, 2016; Xu et al., 2014). These major differences lead us to ask the question of why the *chdA* and *chdB* paralogues have evolved in such a different manner in the two closely related lineages of goldfish and common carp, which shared a genome duplication and were both domesticated. Furthermore, our analyses could not sufficiently explain why the branch length of the goldfish *chdB* gene is longer in comparison with some other *chordin* genes in the phylogenetic tree (Abe et al., 2014, 2016; Figure 1B). More specifically, our molecular developmental genetics led us to conclude that while the *chdA* gene highly contributed to morphological evolution, the nucleotide sequence of *chdB* evolved faster than that of *chdA*, suggesting the conservative nature of *chdA* gene sequence. Several hypotheses can be raised to explain this contradiction between molecular developmental observations and molecular evolutionary processes (e.g., differences between the genes in pleiotropic functions or chromosomal locations); however, this remaining question will require a further detailed study to resolve. We expect that answering above question will provide helpful insights into the mechanisms underlying the retention or loss of paralogous genes and the relationship between molecular evolution and developmental biology in domesticated animals.

## CONFLICTS OF INTEREST

The authors declare that they have no conflicts of interest.

## Supporting information

Supporting information
